# Evidence of shock-compressed stishovite above 300 GPa

**DOI:** 10.1038/s41598-020-66340-y

**Published:** 2020-06-23

**Authors:** Markus O. Schoelmerich, Thomas Tschentscher, Shrikant Bhat, Cindy A. Bolme, Eric Cunningham, Robert Farla, Eric Galtier, Arianna E. Gleason, Marion Harmand, Yuichi Inubushi, Kento Katagiri, Kohei Miyanishi, Bob Nagler, Norimasa Ozaki, Thomas R. Preston, Ronald Redmer, Ray F. Smith, Tsubasa Tobase, Tadashi Togashi, Sally J. Tracy, Yuhei Umeda, Lennart Wollenweber, Toshinori Yabuuchi, Ulf Zastrau, Karen Appel

**Affiliations:** 1 European XFEL, Schenefeld, 22869 Germany; 2 Photon Science, Deutsches Elektronen-Synchrotron DESY, Hamburg, 22607 Germany; 3 Los Alamos National Laboratory, Los Alamos, New Mexico 87545 USA; 4 SLAC National Accelerator Laboratory, Menlo Park, CA 94025 USA; 5 Institute of Mineralogy, Materials Physics and Cosmochemistry, Sorbonne Universités, Paris, 75005 France; 6 RIKEN SPring-8 Center, Sayo-cho, Sayo-gun, Hyogo, 679-5148 Japan; 7 Japan Synchrotron Radiation Research Institute, Sayo-cho, Sayo-gun, Hyogo, 679-5198 Japan; 8 Osaka University, Suita, Osaka 565-0871 Japan; 9 Universität Rostock, Institut für Physik, Rostock, 18051 Germany; 10 Lawrence Livermore National Laboratory, Livermore, CA 94500 USA; 11 Center for High-Pressure Science and Technology Advanced Research (HPSTAR), Shanghai, 201203 China; 12 Earth and Planets Laboratory, Carnegie Institution of Washington, Washington, D.C. 20015 USA

**Keywords:** Planetary science, Astronomy and planetary science, Materials science, Physics

## Abstract

SiO_2_ is one of the most fundamental constituents in planetary bodies, being an essential building block of major mineral phases in the crust and mantle of terrestrial planets (1–10 M_*E*_). Silica at depths greater than 300 km may be present in the form of the rutile-type, high pressure polymorph stishovite (*P4*_2_/*mnm*) and its thermodynamic stability is of great interest for understanding the seismic and dynamic structure of planetary interiors. Previous studies on stishovite via static and dynamic (shock) compression techniques are contradictory and the observed differences in the lattice-level response is still not clearly understood. Here, laser-induced shock compression experiments at the LCLS- and SACLA XFEL light-sources elucidate the high-pressure behavior of stishovite on the lattice-level under *in situ* conditions on the Hugoniot to pressures above 300 GPa. We find stishovite is still (meta-)stable at these conditions, and does not undergo any phase transitions. This contradicts static experiments showing structural transformations to the CaCl_2_, *α*-PbO_2_ and pyrite-type structures. However, rate-limited kinetic hindrance may explain our observations. These results are important to our understanding into the validity of EOS data from nanosecond experiments for geophysical applications.

## Introduction

Stishovite, the high-pressure polymorph of silica, is of vast interest for planetary- and material science as a dominant constituent material in the mantle of Earth and larger terrestrial extra-solar planets. Under equilibrium conditions, stishovite becomes the stable form of SiO_2_ at pressures above ~7 GPa and crystallizes in the rutile-type structure (*P4*_2_/*mnm*), consisting of octahedrally coordinated Si atoms^1–3^. Static compression studies using diamond anvil cell (DAC) techniques show, that stishovite undergoes a displacive phase transition to the orthorhombic CaCl_2_-type structure (*Pnnm*) at ~60 GPa^4–10^ and a further transition to the *α*-PbO_2_ type structure (*Pbcn*) at ~121 GPa^10–16^. To date, the highest-pressure experimentally determined SiO_2_ phase transformation is to the pyrite-type structure (*Pa*$\bar{3}$) at around 268 GPa^17^.

Additionally, silica has been explored using dynamic shock compression experiments. Shock compression studies of fused silica and quartz up to 200 GPa indicate phase transitions to stishovite, post-stishovite phase(s) and melting above 120 GPa^18–23^. However, only few studies use stishovite as a starting material. This is mainly due to the difficulty of synthesizing large specimens of stishovite without impurities or significant porosity, and preparing these samples for shock compression experiments. Stishovite has been shock compressed in the pressure regime between 193.6–235.7 GPa with the gas gun technique^24^, between 316–992 GPa with the flyer plate technique at the Z-machine^25^ and in the pressure regime between 1032.2–2660.4 GPa with the decaying shock method at the OMEGA laser facility^26^. All three studies resolve the stishovite continuum Hugoniot. In contrast to static experiments^5^, there is no indication of a phase transition to post-stishovite structures. This was previously explained by either sluggish kinetics as a result of the low compressibility of stishovite or the relatively small volume changes accompanying post-stishovite phase transitions, which cannot be readily distinguished from the stishovite Hugoniot^24^.

Until now, the highest pressures for which *in*-*situ* structural information of stishovite was obtained are from static compression experiments with a diamond anvil cell at 128 GPa^5^ and there are no structural observations from any shock-compression experiments. The high initial density and low compressibility allowed us to shock-compress stishovite in the solid phase to pressures above 300 GPa. The concurrent use of the brilliant and coherent X-rays at the Linac Coherent Light Source (LCLS) and SPring-8 Angstrom Compact free electron LAser (SACLA), enabled the determination of the structural response under shock loading on the lattice-level of stishovite at these conditions for the first time.

## Results

**Figure 1:**
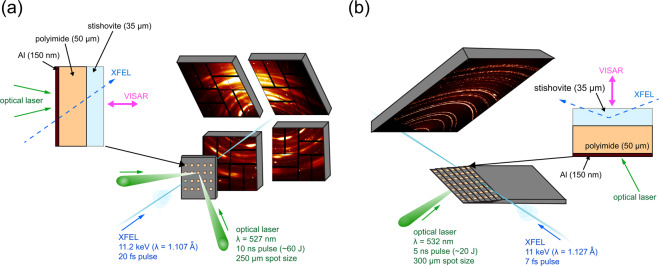
(**a**) Experimental setup arranged in transmission Debye-Scherrer geometry at the MEC end station of the LCLS. Dual drive laser beams were incident on samples at 15° and the XFEL beam at 30° from the target normal. (**b**) Experimental setup in Bragg geometry at the BL3:EH5 of SACLA. Grazing-incidence angle of the XFEL towards the target was 18° and drive laser beam was incident on samples at 18° from the target normal.

The experiments were carried out at the Matter at Extreme Conditions (MEC) end station of the LCLS- and the BL3:EH5 end station of the SACLA X-ray Free Electron Laser (XFEL). The experimental setups are shown in Fig. 1. Polycrystalline stishovite was synthesized in a large volume press at the P61B end station of PETRA III at the German Electron-Synchrotron (DESY) with an initial density of *ρ*_0_ = 4.30 g/cm^3^ (Table 1, run795997). Stishovite samples were cut and polished to 35 μm. Targets were subsequently glued to 50 μm polyimide (kapton) tape and shock compressed using optical drive lasers. Derived from hydrodynamic simulations, steady uniform pressure conditions within the sample are achieved within a time span of ~1–5 ns for the high pressure drives (see Supplementary Material). The samples were probed with an 11.2 keV (LCLS) and 11 keV (SACLA) XFEL X-ray pulse when the majority of it was in the compressed state. This was determined from the shock breakout via the velocity interferometer system for any reflector (VISAR). The VISAR measured the rear free surface velocity of stishovite for each shot from which particle velocity (U_*p*_), shock wave velocity (U_*s*_) and pressure in the shocked state were determined (see Supplementary Material).

**Table 1:** Experimental results from this study.

Run	U_*p*_ (km/s)	U_*s*_ (km/s)	P_*VISAR*_ (GPa)	E-E_0_ (kJ/mol)	V (Å^3^)	*ρ* (g/cm^3^)	a (Å)	c (Å)
SACLA-795997	n.a.	n.a.	ambient	n.a.	46.5 (2)	4.30 (2)	4.176 (8)	2.664 (6)
SACLA-796485	0.45 (2)	9.66 (53)	18 (2)	0.10 (1)	44.3 (3)	4.51 (5)	4.09 (1)	2.65 (1)
SACLA-796491	2.38 (4)	12.10 (25)	123 (5)	2.82 (12)	37.3 (2)	5.35 (4)	3.85 (1)	2.513 (9)
LCLS-235	4.75 (8)	14.76 (31)	301 (12)	11.28 (48)	31.8 (4)	6.3 (1)	3.69 (2)	2.33 (2)
LCLS-233*	4.90 (8)	15.04 (45)	317 (15)	12.01 (56)	31.3 (9)	6.4 (3)	n.a.	n.a.
LCLS-239	5.10 (9)	15.35 (31)	336 (13)	13.01 (51)	31.0 (4)	6.44 (8)	3.66 (2)	2.32 (1)

Particle and shock velocities U_*p*_ and U_*s*_, as well as pressures and energies were determined with the VISAR (see Supplementary Material). Volume, density and lattice parameter *a* and *c* were obtained from refined XRD spectra.

*No XRD information is available for run233. All results from run233 were solely determined through velocimetry data.

The shock and particle velocities (Table 1) obtained in this study yield a linear fit in the high pressure regime

1 $${U}_{s}(km/s)=1.21\,{U}_{p}+9.155$$

and are in excellent agreement with literature data^24^. Pressures P and internal energies E were calculated using the Rankine-Hugoniot equations:

2 $$P-{P}_{0}={\rho }_{0}{U}_{s}{U}_{p}$$

3 $$E-{E}_{0}=\frac{1}{2}(P+{P}_{0})(\mathrm{1/}{\rho }_{0}-\mathrm{1/}\rho )$$

here, E_0_, P_0_ and *ρ*_0_ are reference values at ambient state. Results are listed in Table 1. Pressures obtained from the VISAR agree within 2–6% to EOS data from literature^24^ and density functional theory (DFT) simulations (see Supplementary Table S2).

**Figure 2:**
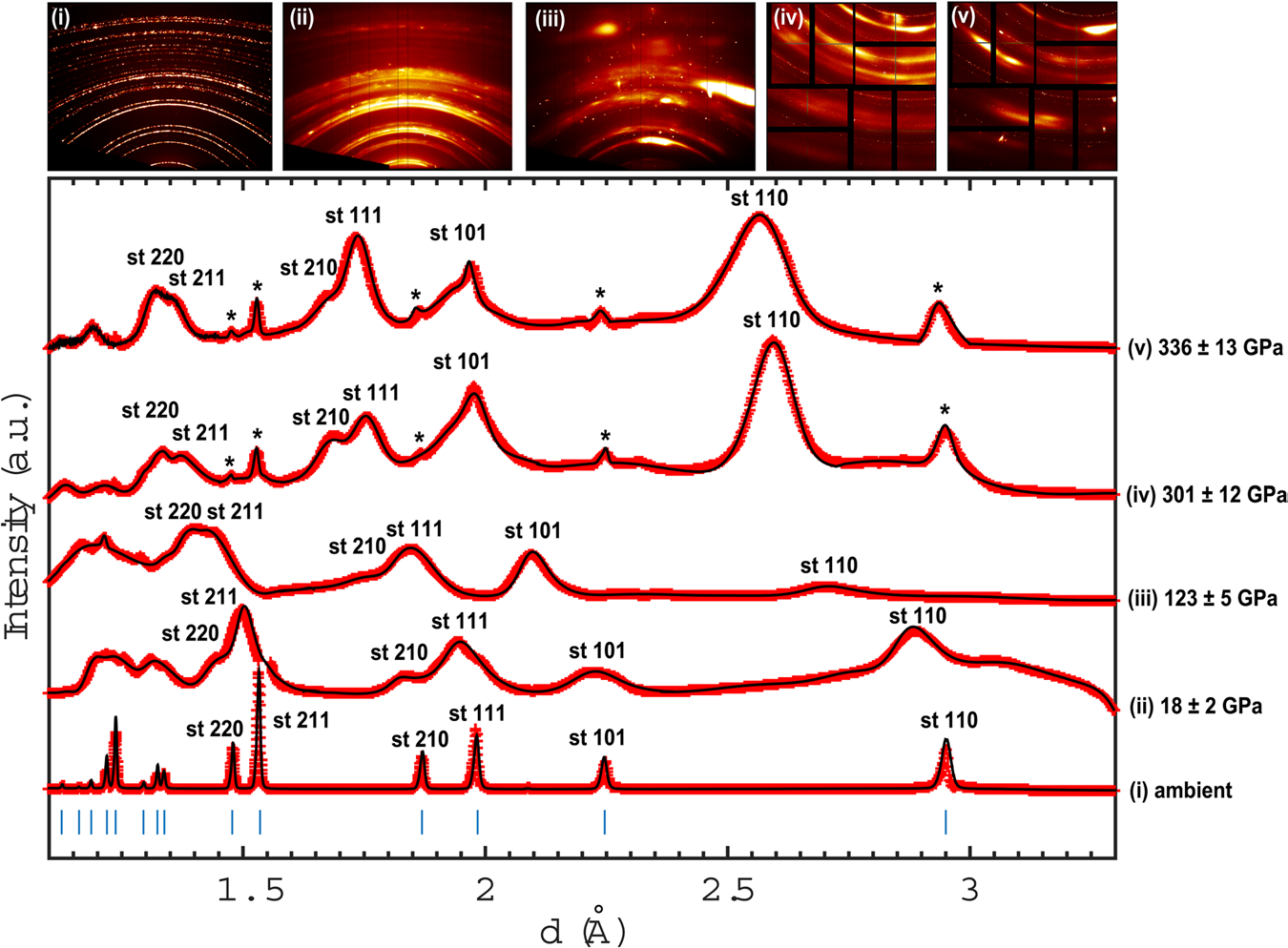
Multiplot of XRD pattern of stishovite. Observed d-spacing (red crosses) and Le Bail-fitted pattern (black line) of stishovite at ambient conditions, 18 ± 2 GPa, 123 ± 5 GPa, 301 ± 12 GPa and 336 ± 13 GPa. Stishovite reflections are indexed at each peak. Ambient peaks are indicated with an asterisk. Note that peaks are shifting to lower d-spacing values due to the change in volume of the lattice with increasing pressure.

Bragg reflections of stishovite are observed upon shock loading up to 336 ± 13 GPa (Fig. 2). The stishovite structure from the refined diffraction pattern at 18 ± 2 GPa reveals a relative change in volume of V/V_0_ = 0.95 at a density of 4.51 g/cm^3^. The achieved pressure is well within the stishovite stability field found in static compression experiments^2,5,10,27–30^. However, a departure from equilibrium behavior is observed at higher pressures: at 123 ± 5 GPa, Bragg reflections of compressed stishovite are still apparent and refined lattice parameters reveal a relative volume change of V/V_0_ = 0.80 at a density of 5.35 g/cm^3^. These conditions are within the equilibrium phase stability field of *α*-PbO_2_ type silica^10–16^. At higher pressures, Bragg reflections of compressed and ambient (marked with an asterisk) stishovite are observed. The refined stishovite reflections at 301 ± 12 GPa indicate a volume change of V/V_0_ = 0.69 at a density of 6.3 g/cm^3^. At 317 ± 15 GPa, density and volume was determined through the VISAR and indicates a relative volume change of V/V_0_ = 0.67 and a shock-density of 6.4 g/cm^3^. At 336 ± 13 GPa, the highest achieved pressure within stishovite in this study, exhibits a volume change of V/V_0_ = 0.66 at a density of 6.44 g/cm^3^.

**Figure 3:**
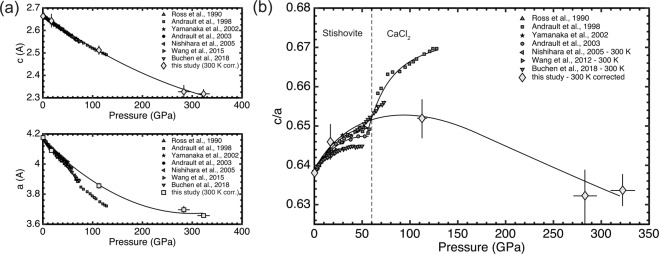
Pressure-density experimental data for stishovite (yellow diamonds) and 300 K corrected data (grey diamonds) from this study and literature. (s) indicates shock wave experiments. Drawn are furthermore the stishovite Hugoniot (dashed line) and 300 K isotherm (solid line).

The pressures, determined from VISAR, as a function of density, obtained from XRD, are shown in Fig. 3. They are in excellent agreement with literature data from shock compression experiments^24–26^ and below 128 GPa comparable to static compression experiments^5,27–30^. We estimated the equation of state (EOS) of stishovite by fitting the data with the EosFit software^31^ to the third-order Birch-Murnaghan equation of state^32^ and, to compare to static data, corrected the shock wave data to a 300 K isotherm. The 300 K isotherm was calculated by subtracting the thermal pressure from the shocked state at fixed volume^27^. We determined the pressure correction by calculating the temperature along the Hugoniot^26^ (see Supplementary Material) and the pressure difference between the Hugoniot and 300 K isotherm using the Grüneisen parameter

4 $$\gamma (V)={\gamma }_{0}{\left(\frac{V}{{V}_{0}}\right)}^{q}$$

with given literature values for q^24^ and *γ*_0_^33^. The shock wave data fit yields a bulk modulus of K_0_ = 307 ± 4 GPa and its first pressure derivative K_0_′ = 4.66 ± 0.15 GPa in the range of 0–336 GPa, which is in excellent accordance to literature data of shock compression experiments (K_0_ = 315 GPa, K_0_′ = 4.8 GPa^18^; K_0_ = 307 GPa, K_0_′ = 5.0 GPa^24^) as well as EOS studies of stishovite at lower pressures from static DAC experiments (K_0_ = 309.9 GPa, K_0_′ = 4.59 GPa^5^; K_0_ = 294 GPa, K_0_′ = 4.85 GPa^27^).

**Figure 4:**
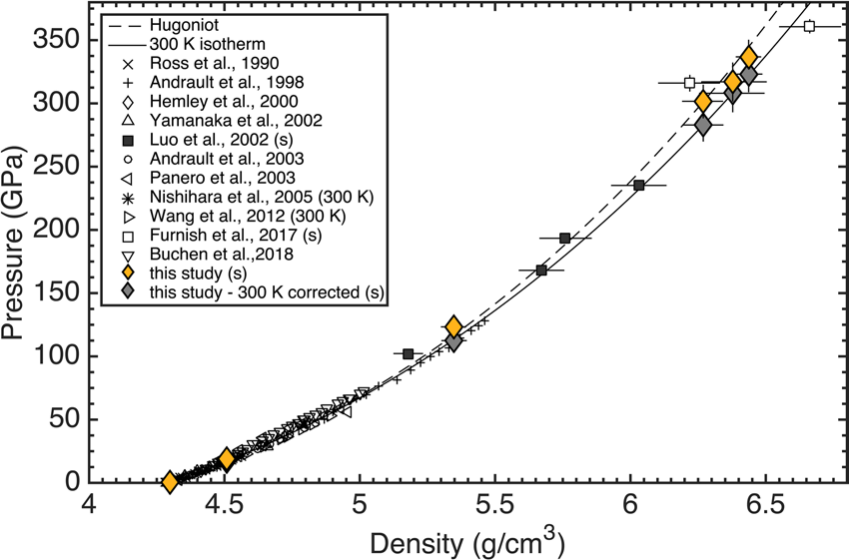
(**a**) Unit-cell parameters *a* and *c* of stishovite with regard to pressure. (**b**) Lattice parameter ratio *c*/*a* with regard to pressure. Indicated is furthermore the pressure, at which a structural transformation from stishovite to CaCl_2_-type silica has been observed in static experiments^5,34^ (dashed line).

Pressure evolution of the SiO_2_ unit-cell parameters from refined XRD pattern are shown in Fig. 4. Here, a deviation from static data^5,34^ is observed for the lattice parameter *a* at pressures exceeding ~60 GPa (Fig. 4a). The lattice parameter ratio *c*/*a* rises with increasing pressures from ~0.638 at ambient conditions to ~0.650 at 123 GPa (Fig. 4b). In this regime the compressibility of the lattice parameter *a* is higher than for lattice parameter *c*. However, at ~123 GPa, this trend is reversing and lattice parameter *c* seems to be more compressible than lattice parameter *a* with a decreasing lattice parameter ratio *c*/*a* from ~0.650 at 123 GPa to ~0.634 at 336 GPa.

## Discussion

**Figure 5:**
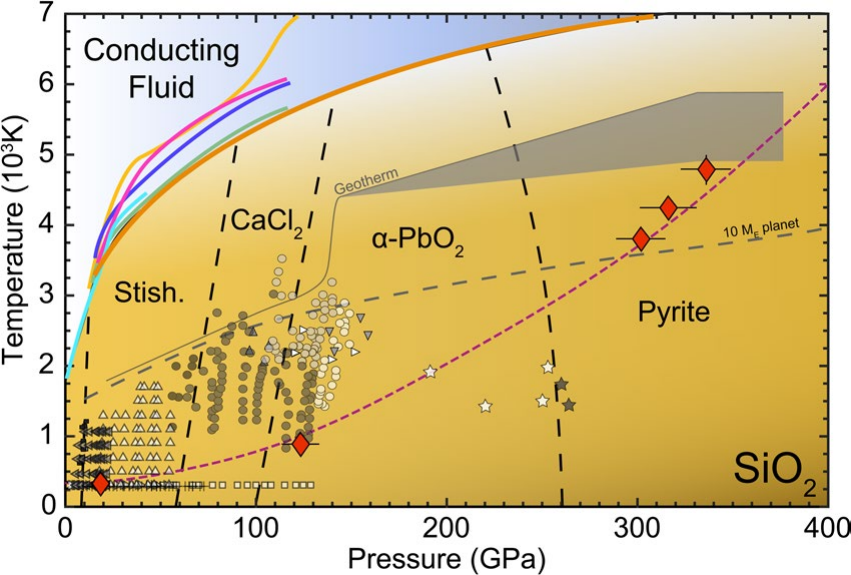
SiO_2_ phase diagram, modified after^18,26,47^. Shown is experimental data where the structure of SiO_2_ was resolved. Indicated are the different equilibrium phase stability fields and the geotherms of Earth and terrestrial planets with a mass of 10 M_*E*_. Red diamonds are data from this study and the dashed magenta line indicates the Sesame stishovite EOS 7360^50^. Shown is data with quartz or fused silica as a starting material: black and grey squares from^2^, circles from^10^, right triangles from^12^, lower triangles from^15^ and stars from^17^ as well as stishovite as a starting material: white squares^4^, upper triangles^27^, crosses^34^ and left triangles^28^. Furthermore melting lines are indicated: brown^26^, orange^51^, pink^52^, blue^53^, green^54^ and light blue^55,56^.

The results of this study on shock compressed stishovite observed by *in*-*situ* X-ray diffraction differ strongly from static compression experiments at respective pressures (Fig. 5). X-ray diffraction studies on stishovite with a DAC show a distinct phase transformation at ~60 GPa to the CaCl_2_ type silica^5,34^. This is, however, in contrast to previous shock compression experiments, where the displacive transition towards CaCl_2_ does not appear in continuum Hugoniot data^24–26^.

Our experimental data is in good accordance with the stishovite EOS of static and shock experiments^5,24^ (Fig. 3) and the bulk moduli from our experiment agree within 1–4% to literature data^5,18,24,27^. It seems that this is not only valid at lower pressures, but continues to be apparent at multi-Mbar pressures^25,26^. Past studies have interpreted the lack of evidence for a phase transition as i) not being in the right pressure regime, ii) sluggish kinetics, or iii) relatively small changes in structure and energy attending a phase transition and not detectable with diagnostics^24^. Up to now, however, there is no lattice-level structural information for stishovite under shock compression. By coupling XFEL sources to high-power laser systems, we can now reveal subtle changes in lattice structure during the shock response of materials. The femtosecond diffraction contradicts i) and iii), since Bragg reflections of stishovite can still be observed in the shock compressed state along the respective Hugoniot at pressures exceeding 300 GPa. Furthermore, the measured lattice parameters do not indicate a change in crystal structure as in a first order transition, in contrast to static data^5,34^ (Fig. 4). The stishovite structure of our shock compression experiments above 300 GPa is therefore in vast contrast to static compression experiments: at 123 GPa, there is no indication of a *α*-PbO_2_ phase, which was seen in DAC experiments with SiO_2_^10,12^ and at experimental peak pressures of 301 GPa to 336 GPa there is no indication of the pyrite structure^17^ at these timescales.

The differences in relative compression between lattice parameter *a* and *c* in our study indicates an anisotropic compression. As predicted by density functional theory, *a* is initially more compressible than *c* but the anisotropic behavior of *a* becomes less pronounced at higher pressure^35^. The relative lattice parameter compression (Fig. 4) even suggests, that this trend eventually reverses and that the polycrystalline stishovite was preferentially orientated within the c-direction during shock transit. Additionally, the broadening of the Bragg reflections in Fig. 2 indicate a pressure gradient and non-hydrostatic stress, with the accuracy of the measured d-values being diminished. It is well known from static (DAC) experiments and molecular dynamic simulations, that (non-) hydrostaticity can fundamentally change the onset, or presence, of phase transitions^36,37^. Previous studies on stishovite in static experiments show, that non-hydrostaticity produces large deviatoric stresses as a result of the high shear strength^7,9^ and it was observed, that the preferred orientation of stishovite under compression is the crystallographic c-direction^38^. However, it was suggested, that non-hydrostatic conditions in DACs actually decrease the pressure-induced onset of a ferroelastic phase transitions from the tetragonal (stishovite) to the orthorhombic (CaCl_2_) structure^8^. This might be different for shock compression experiments in which it is argued, that non-hydrostatic stresses may displace the equilibrium boundaries to higher stresses^39,40^.

In addition to anisotropic compression, due to the absence of phase transformations, shock temperature rises with increasing pressure to adjust to the increase of internal energy due to the compression work^26^. Our calculations indicate, that stishovite is experiencing temperatures ranging from 324 up to 4757 K over a pressure range of 18 to 336 GPa during shock loading (Fig. 5). However, because of the nanosecond timescales during dynamic loading and accommodation of rate-limiting kinetic hinderances, effects can result in significant shifts of equilibrium phase boundaries such that transitions may not be observed or may require significant overpressure. This is a known deviation between shock and static experiments^41–46^. However, the extent to which these pressure induced phase transformations can be hindered seems to be much more pronounced for stishovite than for other materials, presumably due to its high initial density and low compressibility. Our results are unexpected as one would not presume crystallization to be inhibited, at least for the CaCl_2_ structure, which is differentiated from stishovite only by a small diffusionless displacement of oxygen ions.

The pressure and temperature range encompassed in this study is comparable to Super-Earth (1–10 M_*E*_) interior conditions (Fig. 5). However and contrary to our results, it is well established from experimental observation that a stable stishovite structure within the Earth’s mantle at pressures exceeding ~60 GPa is improbable^47^. This can be explained by the short timescales (few ns) involved during our shock compression experiments, which can prevent the observation of long time scale phase transitions as in equilibrated planetary interiors. Indeed, certain phase transitions require at least several nanoseconds to be triggered and can only be observed at longer time scales^20^. Therefore, further time dependence studies under shocked SiO_2_ should be foreseen to bring possible constrains on its physical and chemical properties inside Super-Earth interiors.

In summary, we have determined the shock response of stishovite under shock loading via XRD from the LCLS and SACLA XFEL sources. Additional velocimetry diagnostics confirmed shock data from literature, and provided evidence of the departure from equilibrium behavior of stishovite at pressures exceeding 300 GPa - an unprecedented result. A derived stishovite structure from this study at pressures up to 336 GPa implies a skipping of three different second-order equilibrium phase transitions (CaCl_2_, *α*-PbO_2_ and pyrite-type structure) illustrating how complex structural changes are hindered during dynamic compression experiments.

## Methods

Polycrystalline stishovite was synthesized using the large volume press housed at the PETRA III end station P61B at the German Electron-Synchrotron (DESY). Cylindrical pieces (3 mm in height, 2.5 mm in diameter) were cut from fused silica rods to fit precisely into a Pt-capsule. The Pt-capsule was placed at the center of a 18 mm edge length Cr_2_O_3_ doped MgO octahedral multi-anvil assembly. The octahedral assembly was heated to 1500 K for one hour after compressed to a maximum pressure of 12 GPa (see Supplementary Material). Samples were cut and polished to 35 (±1) μm and glued to 50 μm thick black polyimide (kapton) tape. At LCLS, a Nd:glass optical laser (527 nm, 10 ns, quasi flat top pulse) in combination with 250-μm diameter phase plates was used to launch a smooth and well defined laser pulse onto the targets. At SACLA, a ceramic YAG optical laser (532 nm, 5 ns, quasi flat top pulse) in combination with 300-μm diameter phase plates was applied. Detailed information about the experimental platforms can be found in the references^48,49^. Five different maximum drive laser energies were used for the experiments: 19.1 J and 22.1 J at SACLA, and 48.6 J, 50.6 J, 51 J at LCLS. Free surface velocities were recorded through the VISAR and shock velocities were calculated from velocimetry and diffraction data (see Supplementary Material). Two VISAR legs where employed to resolve fringe-jump ambiguity from the compressed sample velocity traces. The LCLS and SACLA FEL sources provided quasi-monochromatic (dE/E = 0.2–0.5%), coherent, 11.2 keV (LCLS) and 11 keV (SACLA) X-ray pulses of ~20 fs (LCLS) and ~7 fs (SACLA) duration. The XFEL beam at LCLS was focused to 50 × 50 μm^2^ onto the target and centered to the focal spot of the drive laser. At SACLA, the XFEL beam was focused to 10 × 30 μm^2^. The LCLS experiment was conducted in transmission geometry normal to the target whereas the SACLA experiment was conducted in reflection geometry with a grazing-incidence angle of 18° between sample surface and XFEL beam (Fig. 1). The XFEL probed the samples at shock breakout (±0.5 ns), hence when the sample was completely compressed. Four Cornell-SLAC Pixel Array Detectors (CSPADs) at LCLS and a large flat panel detector (CMOS camera coupled with an X-ray scintillator) at SACLA recorded the resulting diffraction from the XFEL probe beam during shock compression. The diffraction images were azimuthally integrated and are shown as XRD pattern in Fig. 2.

## Supplementary information

Supplementary Information.
