# Supplementary material for: Evidence of shock-compressed stishovite above 300 GPa
Source: Sci Rep. 2020 Jun 23;10:10197. doi: 10.1038/s41598-020-66340-y (PMC7311448; doi:10.1038/s41598-020-66340-y)
Supplement: Supplementary file 1 — Supplementary Information. [file 41598_2020_66340_MOESM1_ESM.pdf]

Evidence of shock-compressed stishovite above 300 GPa

Markus O. Schoelmerich[1*], Thomas Tschentscher[1], Shrikant Bhat[2], Cindy A. Bolme[3], Eric Cunningham[4], Robert Farla[2], Eric Galtier[4], Arianna E. Gleason[4], Marion Harmand[5], Yuichi Inubushi[6,7], Kento Katagiri[8], Kohei Miyanishi[6], Bob Nagler[4], Norimasa Ozaki[8], Thomas R. Preston[1], Ronald Redmer[9], Ray F. Smith[10], Tsubasa Tobase[11], Tadashi Togashi[6,7], Sally J. Tracy[12], Yuhei Umeda[8], Lennart Wollenweber[1], Toshinori Yabuuchi[6,7], Ulf Zastrau[1], and Karen Appel[1]

[1]European XFEL, 22869 Schenefeld, Germany
[2]Photon Science, Deutsches Elektronen-Synchrotron DESY, 22607 Hamburg, Germany
[3]Los Alamos National Laboratory, Los Alamos, New Mexico 87545, USA
[4]SLAC National Accelerator Laboratory, Menlo Park, CA 94025 USA
[5]Institute of Mineralogy, Materials Physics and Cosmochemistry, Sorbonne Universités, Paris, 75005, France
[6]RIKEN SPring-8 Center, Sayo-cho, Sayo-gun, Hyogo 679-5148, Japan
[7]Japan Synchrotron Radiation Research Institute, Sayo-cho, Sayo-gun, Hyogo 679-5198, Japan
[8]Osaka University, Suita, Osaka, 565-0871 Japan
[9]Universität Rostock, Institut für Physik, Rostock, 18051, Germany
[10]Lawrence Livermore National Laboratory, Livermore CA 94500, USA
[11]Center for High-Pressure Science and Technology Advanced Research (HPSTAR), Shanghai 201203, China
[12]Earth and Planets Laboratory, Carnegie Institution of Washington, Washington, D.C. 20015, USA
*markus.schoelmerich@xfel.eu

SUPPLEMENTARY MATERIAL

Synthesis of polycrystalline stishovite

Polycrystalline stishovite was synthesized in an 18/10 assembly using the large volume press housed at the PETRAIII end station P61B at the German Electron-Synchrotron (DESY). Cylindrical rods of fused silica with a diameter of 2.5 mm were cut and polished to 3 mm in height, cleaned and inserted into a Pt-capsule. The capsule was placed into an MgO gasket with MgO spacer on top and bottom. The MgO was then surrounded by an $LaCrO_3$ furnace to heat the sample during the experiment. Molybdenum discs and rods between ZrO_2 spacer were used for propagation of the current towards the furnace. The assembly was then inserted into an 18 mm edge length Cr_2O_3-doped MgO octahedron (Fig. S1). The octahedron was put into the center of eight WC-cubes with a truncation of 10 mm and subsequently compressed in the LVP. The octahedral assembly was heated to 1500 K for one hour after compressed to a maximum pressure of 12 GPa. Pressure was calibrated at room temperature using the semiconductor to metal transition of ZnTe at 9.6 GPa and 12 GPa. Sample temperatures were estimated using power-temperature relations calibrated in a separate run using a W5%Re/W26%Re thermocouple (C-type).

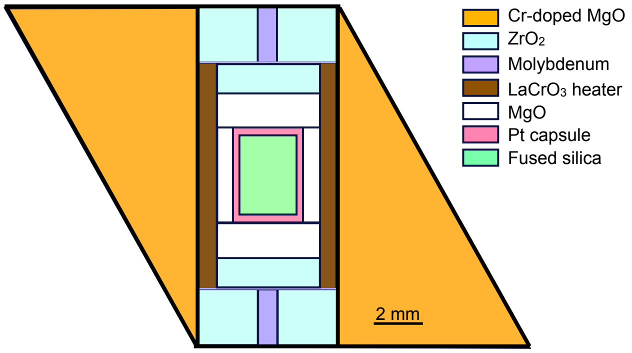

Figure S 1. Experimental assembly of a large volume press experiment used for the synthesis of polycrystalline stishovite.

Characterization and preparation of the synthesized stishovite

The synthesized products were translucent cylinders with no visible cracks or impurities (Fig. S2). The run products were analyzed with a bench-top X-Ray diffractometer (Europe 600, GNR Analytical Instrument Group) at the P61B end station (Cu - Kα; λ = 1.5418 Å). Diffraction of the products confirmed the pure stishovite phase composition. Grain size of the stishovite was determined through XRD with the commonly used Scherrer equation[1]

$$\tau = \frac{K\lambda}{\beta cos\theta} \tag{1}$$

with τ = grain size, K = shape factor (commonly set to 0.94), λ = X-ray wavelength, β = line broadening at full width at half maximum (FWHM) corrected of the instrumental broadening and θ = Bragg angle. Typical average grain size of the synthesized samples was ~0.52(2) μm. After the experiment, stishovite cylinders were cut to 2-3 discs (around 300 μm thick, ~2 mm diameter) with a diamond saw. The discs were then polished from both sides down to ~35 μm.

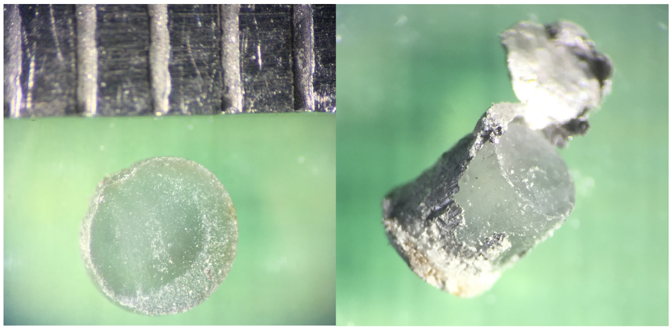

Figure S 2. Experimental stishovite run products from the synthesis. The stishovite cylinder on the right is still encapsulated in platinum.

XRD refinement

Using the EXPO2014 software[2] we performed Le Bail refinement on the integrated XRD diffraction data of the ambient (run795997) and shock compressed stishovite. The refinement was done by using the known stishovite structure[3]. The preferred profile function was the Pearson VII peak shape function. The lattice parameters (a,c), unit cell volume (V), density (ρ), goodness of fit factor (χ^2) and the reliability factors, R_{wp} and R_p are listed in supplementary Table 1. We note, that larger χ^2 factors are seen for the shock-compressed runs at LCLS, due to the partially ambient phase and the gaps of the CSPADs. To calibrate the sample to detector distances and detector tilts, we used the NIST Standard Reference Materials (SRM) CeO_2 (674b, LCLS) and LaB_6 (660a, SACLA).

**Stishovite**							
Run	Volume (Å^3)	a(Å)	b (Å)	c (Å)	reduced-χ^2	R_{wp}	R_p
795997	46.5(2)	4.176(8)	n.a.	2.664(6)	1.153	2.474	1.689
796485	44.3(3)	4.09(1)	n.a.	2.65(1)	1.422	3.724	2.372
796491	37.3(2)	3.85(1)	n.a.	2.513(9)	1.367	2.473	1.602
235	31.8(4)	3.69(2)	n.a.	2.33(2)	3.590	2.615	1.917
239	31.0(4)	3.66(2)	n.a.	2.32(1)	1.962	3.661	2.526
**CeO_2**							
57	158.49(2)	5.4(5)	n.a.	n.a.	1.244	5.802	3.427
**LaB_6**							
795425	70.53(2)	4.1(7)	4.14(4)	4.13(3)	1.092	2.493	1.670

Table S 1. The lattice parameters (a,c), the unit-cell volume (V), the goodness of fit factors (reduced-χ^2) and the reliability factors, R_{wp} and R_p, derived from Le Bail refinements of stishovite and the standard materials CeO_2 and LaB_6.

Pressure determination

The VISAR recorded the free surface velocity U_{fs} and reflectivity of the shocked stishovite samples (Fig. S3), which can be converted to particle velocity U_p by using the acoustic approximation $U_{fs} = 2U_p$. The shock velocity U_s was determined by i) measuring the transit time of the shock wave (the change of reflectivity) through our sample with well known thickness and calculating U_s by dividing the thickness of our sample with the shock wave transit time ($U_s = d_{sample}/t_{transit}$) and ii) with the help of our diffraction measurements by using the Rankine-Hugoniot relationship

$$U_s = \frac{U_p}{1 - \rho_0/\rho} \tag{2}$$

U_p was measured by VISAR whereas ρ_0 (initial density) and ρ (shocked density) was determined through our refined diffraction measurements. Methods i) and ii) were in very good agreement with differences from each other typically at ∼1 %.

We could then determine the peak sample pressure using

$$P = \rho_0 U_s U_p \tag{3}$$

Uncertainties of the VISAR measurements were typically between 10-15 % and arise predominantly from the uncertainties of the free-surface velocity profile (Fig. S3). Additionally, the density measurements of the refinement held an uncertainty due to the fitting error of the diffraction data. The uncertainty from XRD was, however, much smaller than from VISAR measurements, typically between 1-2 %.

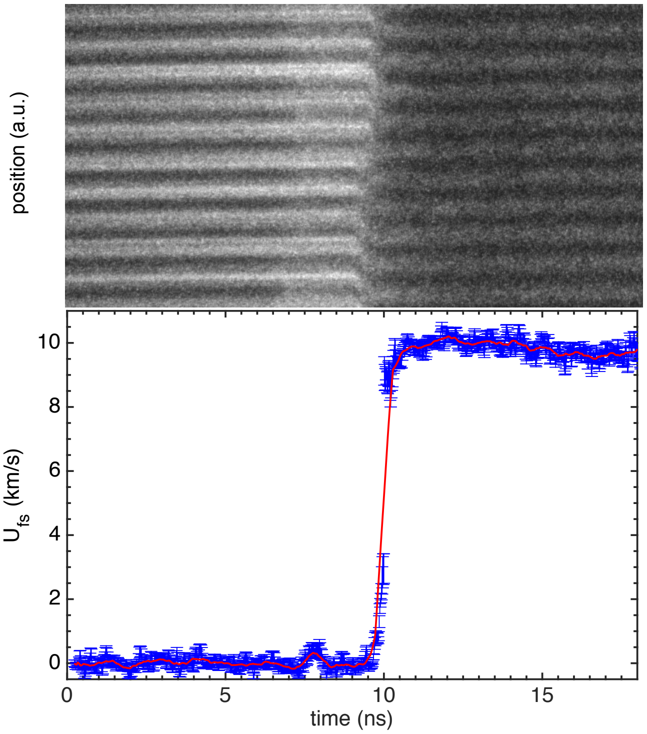

Figure S 3. Typical VISAR image from the experiment and the corresponding free surface velocity trace (bottom image, red line). Furthermore, standard deviation of the velocity are shown (blue error bars).

A linear fit to the U_s-U_p relationship from this study in the high pressure stishovite regime is shown in Figure S4. Note, that U_s for run233 was solely calculated by dividing the sample thickness with the shock transit time, which was determined from the reflectivity change in the VISAR image.

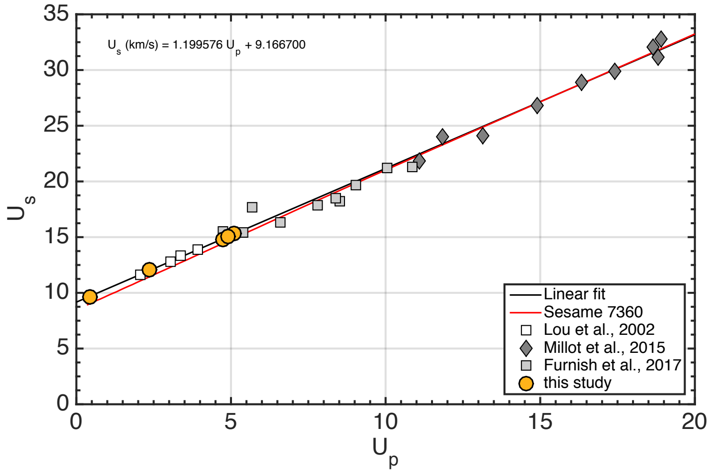

Figure S 4. U_s - U_p relationship from this study and literature[4–7].

Determination of the temperature along the Hugoniot

Estimation of the reached temperature conditions of the individual experimental runs were done according to the approach of[7], using the Mie-Grüneisen formalism[8,9]

$$\gamma(V) = V\frac{\partial P(V,T)}{\partial E(V,T}|_V \tag{4}$$

The Grüneisen parameter γ(V) is assumed to be only a function of the volume

$$\gamma(V) = \gamma_0\left(\frac{V}{V_0}\right)^q ; \gamma_0 = 1.35 \tag{5}$$

with q = 2.6[5], V_0 as the ambient specific volume and γ_0 the ambient Grüneisen parameter[10]. Change of V/V_0 is shown in Figure S5.

The pressure on the isentrope can be derived from the Eulerian strain framework[11], using a finite strain parameter f

$$f = \frac{1}{2}\left[\left(\frac{V_0}{V}\right)^{\frac{2}{3}} - 1\right] \tag{6}$$

and the third-order Birch-Murnaghan strain-stress relation[12]

$$P_S = 3K_{0,S}f(1+2f)^{\frac{5}{2}}[1+(3/2)(K'_{0,S}-4)f] \tag{7}$$

Furthermore, the internal energy of the isentrope can be described through

$$\Delta E_S = (9/2)V_0K_{0,S}f^2[1+(K'_{0,S}-4)f] \tag{8}$$

To calculate the shock temperature, we can use the Debye model using the Debye temperature θ(V):

$$\theta(V) = \theta_0\left(\frac{V_0}{V}\right)^{\gamma} \tag{9}$$

and the specific heat C_V as a function of the temperature only:

$$C_V(T) = 9nR\left(\frac{T}{\theta_0}\right)^3 \int_0^{\theta_0/T} \frac{x^4 e^x}{(e^x-1)^2} dx \tag{10}$$

with n the number of atoms in a formula unit (n=3, SiO_2) and R being the perfect gas constant.

From equation 4 and 5 we can write

$$P_H - P_S = \frac{\gamma}{V}(E_H - E_S) \tag{11}$$

with H being the states along the Hugoniot and S for the isentrope, derived from the reference state E_0, V_0 and T_0.

The Rankine-Hugoniot relation gives:

$$E_H = E_0 + \frac{1}{2}(P_H + P_0)(V_0 - V) \tag{12}$$

Using equation 4, 5 and 12, we obtain the pressure along the Hugoniot:

$$P_H = \frac{P_S - \gamma(\frac{E_S}{V} - \frac{P_0}{2})(\frac{V_0}{V} - 1)}{1 - \frac{\gamma}{2}(\frac{V_0}{V} - 1)} \tag{13}$$

With the equations 4, 5, 7 and 12 we can write:

$$\frac{V}{\gamma}(P_H - P_S) = \int_{T_H}^{T_S} C_V dT \tag{14}$$

With

$$dS = \frac{\gamma C_V}{V} dV + \frac{C_V}{T} dT \tag{15}$$

and

$$ln\left(\frac{T_S(V)}{T_0}\right) = -\int_V^{V_0} \frac{\gamma}{V} dV \tag{16}$$

we can calculate the temperature along the isentrope using equation 5 as

$$T_S(V) = T_0 exp\left(\frac{\gamma_0)}{q}\left[1 - \left(\frac{V_0}{V}\right)^q\right]\right) \tag{17}$$

The temperature along the Hugoniot can then be calculated by solving equation 14 using a constant specific heat C_V = m(3nR) with m being a multiplier to the Duong-Petit limit value:

$$T_H = T_S + \frac{V)}{m(3nR)\gamma}(P_H - P_S) \tag{18}$$

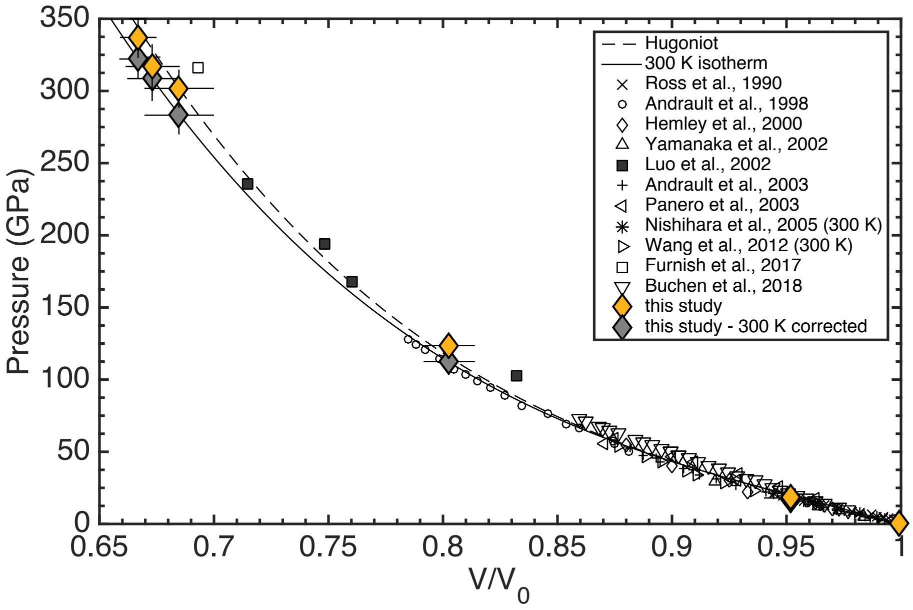

Figure S 5. Relative volume change with regard to pressure from this study and literature.

Pressure comparison to literature data and DFT simulations

In order to compare our results to first principle simulations, we computed the ground states from density functional theory (DFT) starting with the refined structures and densities of our experiments. DFT is implemented in the Quantum ESPRESSO package[13] solving the Kohn-Sham equations self consistently to minimize the free energy of the system as a functional of the local electron density. We used the exchange-correlation functional of Perdew, Burke and Ernzerhof (PBE), which has been shown to give reasonable results in the warm dense matter regime and the projected augmented waves (PAW) pseudopotential approach. We performed DFT using 48 atoms in a 2×2×2 tetragonal supercell. We used Γ centered k-points to sample the entire Brillouin zone. The effect of the finite size in the simulation zone cell was investigated by using a larger cell of 384 atoms (3×3×3), however no immediate effect on the internal stress was observed. A good compromise between accuracy and computation speed was found for the plane-wave expansion of the Kohn-Sham orbitals with a cutoff of 80 Ry.

The determined pressures from DFT are in reasonable agreement to our study and to literature data (Tab. S2). We did not compute the internal stress for run233, since a refined starting structure was not available.

Run	P_{VISAR} (GPa)	P_{EOS}[5] (GPa)	P_{DFT} (GPa)
796485	18(2)	17.1	20.6
796491	123(5)	119.3	118.9
235	301(12)	313.8	305.1
233*	317(15)	329.6	n.a.
239	336(13)	359.1	335.9

Table S 2. Pressure comparison to literature data and density functional theory.

Bragg reflections of stishovite obtained in this study compared to high pressure SiO_2 polymorphs

We calculated theoretical Bragg reflections of the stishovite, PbO_2 and pyrite-type silica structures at 336 GPa and plotted the XRD spectra of run239 in Figure S6 in order to identify respective XRD peaks. No indication of other phases, except stishovite, are observed.

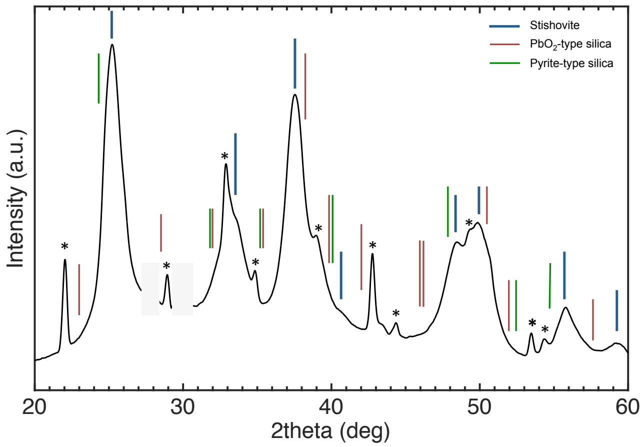

Figure S 6. XRD outline (black line) from run235 at 301 GPa. Indicated are Bragg Peaks of stishovite (blue lines), of the PbO_2 type silica (red line) and the pyrite-type SiO_2 (green line). Peaks with an asterisk indicate still un-shocked stishovite. Gray areas indicate discontinuities in the plot from the detector gaps.

d-spacing of stishovite from this study compared to calculated high pressure SiO_2 polymorphs

We compared *d*-spacing positions of the high-pressure polymorphs of SiO_2 at obtained pressures from this study, to rule out the $CaCl_2$, α -PbO_2 and pyrite-type silica structures. Peak splitting of the $CaCl_2$ structure is not observed in the XRD spectra. Furthermore, *d*-spacing of α -PbO_2 and pyrite-type silica does not fit obtained XRD spectra from this study.

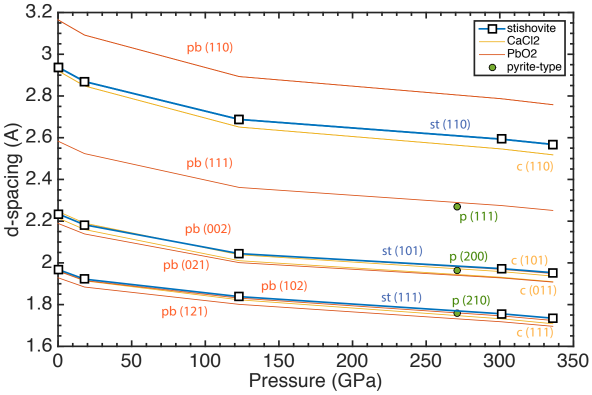

Figure S 7. Measured *d*-spacing of primary stishovite reflections at given pressures (squares) compared to calculated *d*-spacing positions of stishovite (blue), $CaCl_2$ (yellow), α -PbO_2 (red) and pyrite-type silica (green dots).

Hydrodynamic simulations of the experimental conditions

We carried out hydrodynamic simulations of the experiment using the hydrodynamic code HELIOS[14] to constrain experimental conditions. For the simulations, we used a 10 ns top hat pulse shape at a wavelength of 527 nm and a laser energy of 51 J. Sample sequence was 50 μm kapton tape and 35 μm stishovite. The simulations employed the SESAME 7360 equation of state for stishovite.

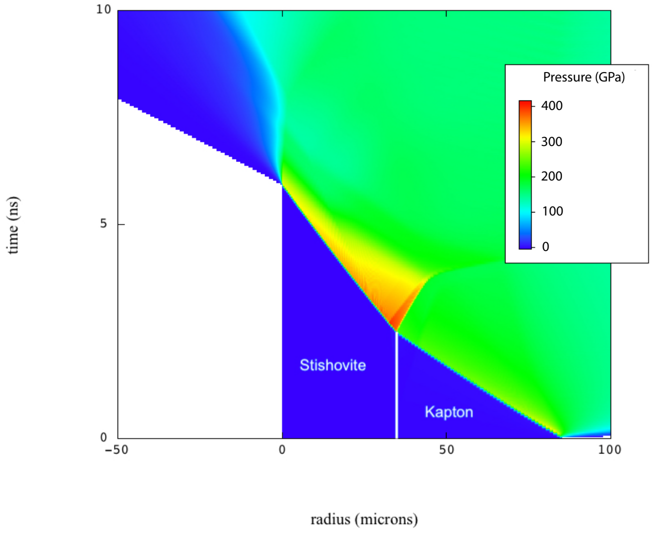

Figure S 8. Pressure conditions derived from hydrodynamic simulations using the hydrodynamic code HELIOS[14].

References

1. Langford, J. I. & Wilson, A. Scherrer after sixty years: a survey and some new results in the determination of crystallite size. *J. applied crystallography* **11**, 102–113 (1978).
2. Altomare, A. *et al.* Expo2013: a kit of tools for phasing crystal structures from powder data. *J. Appl. Crystallogr.* **46**, 1231–1235 (2013).
3. Ross, N. L., Shu, J. & Hazen, R. M. High-pressure crystal chemistry of stishovite. *Am. Mineral.* **75**, 739–747 (1990).
4. Kerley, G. Equations of state for composite materials. *Rep. KPS99* **4** (1999).
5. Luo, S.-N., Mosenfelder, J. L., Asimow, P. D. & Ahrens, T. J. Direct shock wave loading of Stishovite to 235 GPa: Implications for perovskite stability relative to an oxide assemblage at lower mantle conditions. *Geophys. Res. Lett.* **29**, 36–1–36–4 (2002).
6. Furnish, M. D., Shulenburger, L., Desjaralais, M. & Fei, Y. Recent research on stishovite: Hugoniot and partial release Z experiments and DFT EOS calculations (with nov. 17 2017 reanalysis). Tech. Rep., Sandia National Lab.(SNL-NM), Albuquerque, NM (United States) (2017).
7. Millot, M. *et al.* Shock compression of stishovite and melting of silica at planetary interior conditions. *Science* **347**, 418–420 (2015).
8. Jeanloz, R. *et al.* Achieving high-density states through shock-wave loading of precompressed samples. *Proc. Natl. Acad. Sci.* **104**, 9172–9177 (2007).
9. Fei, Y., Mao, H.-k. & Hemley, R. J. Thermal expansivity, bulk modulus, and melting curve of H_2O–ice VII to 20 GPa. *The J. chemical physics* **99**, 5369–5373 (1993).
10. Watanabe, H. *et al.* Thermochemical properties of synthetic high-pressure compounds relevant to the Earth's mantle. *High-pressure Res. Geophys.* 441–464 (1982).
11. Meade, C. & Jeanloz, R. Static compression of $Ca(OH)_2$ at room temperature: observations of amorphization and equation of state measurements to 10.7 GPa. *Geophys. Res. Lett.* **17**, 1157–1160 (1990).
12. Birch, F. Finite strain isotherm and velocities for single-crystal and polycrystalline NaCl at high pressures and 300 K. *J. Geophys. Res. Solid Earth* **83**, 1257–1268 (1978).

13. Giannozzi, P. *et al.* Quantum espresso: a modular and open-source software project for quantum simulations of materials. *J. physics: Condens. matter* **21**, 395502 (2009).

14. MacFarlane, J., Golovkin, I. & Woodruff, P. Helios-cr–a 1-d radiation-magnetohydrodynamics code with inline atomic kinetics modeling. *J. Quant. Spectrosc. Radiat. Transf.* **99**, 381–397 (2006).
